# Interprofessional perspectives on the implementation of a pilot graduate nurse residency program in a Hungarian pediatric hospital

**DOI:** 10.3389/fmed.2026.1833767

**Published:** 2026-09-14

**Authors:** Edit Nyergesné Závodi, Viktor Simon, Krisztina Antónia Bornemissza, Elek Fazekas, Borbála Mikos, György János Velkey, Helga Judit Feith

**Affiliations:** 1 Doctoral College Health Sciences Division, Semmelweis University, Budapest, Hungary; 2 Bethesda Children’s Hospital, Budapest, Hungary; 3 Faculty of Health Sciences and Social Studies, Károli Gáspár University of the Reformed Church in Hungary, Budapest, Hungary; 4 Faculty of Humanities and Social Sciences, Pázmány Péter Catholic University, Budapest, Hungary; 5 Department of Social Sciences, Faculty of Health Sciences, Semmelweis University, Budapest, Hungary

**Keywords:** attitude survey, graduate nurse residency program, implementation, nursing shortage, professional development, nursing education

## Abstract

**Background:**

Ensuring nursing workforce supply and strengthening retention are critical healthcare challenges. Bethesda Children's Hospital introduced a pilot graduate nurse residency program (GNRP) to support newly graduated nurses through structured mentoring, rotation, and educational support. This study examines healthcare professionals' perceptions and acceptance of the program, rather than its effectiveness.

**Methods and sample:**

A cross-sectional, quantitative, anonymous questionnaire survey was conducted at Bethesda Children's Hospital. The survey assessed sociodemographic and professional characteristics, attitudes toward the GNRP, and opinions on key program elements. Data were analyzed using descriptive statistics, analysis of variance, factor analysis, and regression analysis (IBM SPSS Statistics 27). Of 394 eligible employees, 189 participated (84 physicians, 44%; 105 nurses, 56%), representing occupational roles adequately (*χ*^2^ = 0.041; *p* = 0.839).

**Results:**

Perceived support was generally positive, with mean ratings of program elements ranging from 3 to 4 on a 4-point Likert scale and low standard deviations (0.5-0.8). Factor analysis identified two dimensions: overall program support and ward-level mentoring support. Physicians reported significantly higher overall support than nurses (*p* < 0.001), whereas no significant professional difference was found in ward-level support (*p* = 0.542). Regression analysis showed that overall support was associated with professional role and ward-level support (*p* < 0.001), while years spent working in healthcare was not an independent factor.

**Conclusions:**

The GNRP was perceived favorably in this Hungarian pediatric hospital, although nurses expressed more reserved support than physicians. This pilot study provides initial empirical evidence on the acceptance and perceived feasibility of introducing a graduate nurse residency program in Hungary.

## Introduction

Shortage of healthcare professionals is one of the most pressing challenges for the sustainability of healthcare systems (1). Within this context, the issue of nursing shortages is particularly prominent (2), with severe consequences for the quality, accessibility, and safety of patient care both globally and regionally (3). According to the 2025 report of the World Health Organization, although the number of nurses has been increasing worldwide in recent years, substantial regional disparities remain in Europe, with a global shortage of 4.1 million nurses projected by 2030 (4). In Europe, approximately 1.2 million physicians, nurses, and midwives are currently lacking, with considerable regional differences (5). In Hungary, for example, there were only 6.8 nurses per 1,000 inhabitants in 2024, compared with 10 per 1,000 in Denmark and 12.25 per 1,000 in Germany. The EU average was 8.5 per 1,000 inhabitants (6, 7). Hungary is located in the lower third of the European ranking, which causes serious challenges for the long-term sustainability of healthcare provision (8). Shortage results from complex structural factors, while demographic changes and demand for healthcare services continue to increase (2). The literature indicates that low nurse-to-patient ratios are directly associated with higher mortality rates, more frequent patient safety events, and higher incidence of hospital-acquired infections (9, 10). Excessive workload for existing staff leads to exhaustion, job dissatisfaction, and workforce attrition, further exacerbating the situation (11, 12).

One of the main causes of nursing shortage is high turnover, which poses a serious problem worldwide. In a comprehensive 2024 analysis, Ren and colleagues processed data from 14 countries and found that the average nursing turnover rate was 16% (13). A systematic review conducted on a broad sample produced similar findings, suggesting that turnover is not an isolated problem but a phenomenon affecting healthcare systems worldwide, threatening their proper functioning (14).

The beginning of professional careers for newly graduated nurses is an extremely critical and stressful period, often described in the literature as transition shock (15). According to a 2023 study, early-career nurses are particularly prone to low job satisfaction and inadequate stress management, along with insufficient support, which further intensifies the severity of transition shock during their first year (15, 16). Newly graduated nurses often face critical situations for which they do not feel adequately prepared; these situations can cause high levels of anxiety, uncertainty, and frustration, and may increase the risk of leaving the profession (17, 18). International studies emphasize that inadequate support and structured transition programs can exacerbate difficulties experienced by newly graduated nurses during transition from formal education to the workplace. Professional mentoring and graduate nurse residency programs contribute significantly to successful adaptation, development of clinical skills, professional integration, and reduction of psychological stress (19). In addition, a supportive work environment, regulated workload, and constructive feedback are essential for retaining newly graduated nurses (20, 21).

The concept of the graduate nurse residency program (GNRP) emerged in the early 2000s to facilitate the integration of newly graduated nurses from education into clinical practice. In this manuscript, the term GNRP is used to refer to structured residency-type transition programs for newly graduated nurses. To our knowledge, the first program was established in the United States by the University of Colorado College of Nursing in collaboration with the University Health System Consortium and the American Association of Colleges of Nursing (AACN) (22). This pioneering model, first implemented at the University of Colorado Hospital, established the national standard for postgraduate nurse residency training in the United States (23). The effectiveness of such programs has been confirmed by numerous systematic reviews, demonstrating that these programs can improve retention rates during the first 12 months. In a meta-analysis, a 96% retention rate was observed among newly graduated nurses participating in the study (24, 25).

In general, these programs can be regarded as important educational and organizational interventions that facilitate professional integration of newly graduated nurses, development of their clinical competence, and workforce retention within the healthcare system (25, 26).

Hungary belongs to the group of European countries with lower nursing density: in 2021, it had only 6.59 nurses per 1,000 inhabitants, well below the EU average, which was substantially higher according to OECD data (e.g., EU median: ∼8-9 per 1,000 inhabitants) (27). In response to domestic challenges, Bethesda Children's Hospital played a pioneering role in 2023 by launching Hungary's first graduate nurse residency program (GNRP). The two-year program is designed for newly graduated nurses and combines three-month rotations across patient care units, professional mentoring by experienced nurses, psychological mentoring, and regular theoretical continuing education. The implementation process includes the recruitment and orientation of residents, allocation to clinical areas, mentor assignment, scheduled rotations, and periodic professional feedback. The program aims to strengthen professional integration, reduce early-career uncertainty, support career development, and adapt international residency practices to the Hungarian pediatric hospital context through a complex system of professional and psychosocial support (28). Accordingly, the aim of this pilot study was to examine how physicians and nurses perceive and accept the implementation of the first GNRP in Hungary.

## Methods and sample

For the pilot program to be successful, it is important that it is accepted by the professional community at international, national, and local levels. As this type of program was launched in Hungary for the first time, a self-developed questionnaire-based survey was conducted among the affected population to assess individual elements of the program and to evaluate healthcare professionals' acceptance of the GNRP as a whole.

The questionnaire was administered among nurses and physicians at Bethesda Children's Hospital. The GNRP was developed and implemented by the hospital's nursing leadership and professional management; therefore, the study explicitly focused on employees' perceptions and acceptance of an institutionally developed initiative. Completed questionnaires were collected anonymously in sealed envelopes, and the analysis was conducted using anonymized data. The research process was approved by the Scientific and Research Ethics Committee of the Medical Research Council (file number: BM/23058-3/2024). As this was the first pilot study conducted in this context, it was exploratory in nature and examined perceived support for the program, its evaluation, and various components of the comprehensive program in relation to background variables. Data were collected using a self-developed questionnaire. The study employed a cross-sectional, quantitative design and was conducted as an anonymous, voluntary questionnaire survey.

The questionnaire focused on demographic and professional characteristics of participants, as well as their evaluations and opinions regarding program elements. The self-developed questionnaire included Likert scales assessing program elements and general attitudes. The scales ranged from 1 to 4, where respondents indicated the extent to which they agreed with or considered a given program element appropriate, with higher values reflecting more positive evaluations of the program. A four-point Likert scale was selected to avoid a neutral midpoint response and to encourage respondents to indicate a more clearly positive or negative position regarding the program. In terms of scale construction, two types of Likert scales were developed: the first assessed the program from the perspective of resident nurses, while the second examined opinions regarding the training and organizational support components of the program. To measure whether respondents considered the program useful for the professional development of nurses, we used a 4-point Likert scale. Higher values generally indicated more favorable opinions.

Descriptive statistics, analysis of variance, and cluster analysis were applied to analyze data, enabling the identification of distinct supportive attitudes. In relation to attitudes toward the program, exploratory factor analysis (principal axis factoring) was conducted as the first step using the IBM SPSS Statistics 27 software package. Suitability of data was confirmed by the Kaiser–Meyer–Olkin measure (KMO = 0.847) and Bartlett's test [*χ*^2^ (45) = 861.67; *p* < 0.001].

The target population of the study consisted of nurses and physicians at Bethesda Children's Hospital. According to the human resources department of the hospital, the eligible staff included 216 nurses (55%) and 178 physicians (45%), resulting in a total eligible population of 394 employees. All eligible employees were invited to participate in the anonymous survey. The sample comprised 189 participants, corresponding to a response rate of 48.0%; 84 respondents were physicians (44.44%), and 105 were nurses (55.56%). Because participation was anonymous and voluntary, no separate register of explicit refusals was kept; non-returned questionnaires were treated as non-response. Based on the Chi-square test, the sample was representative with respect to occupational roles (*χ*^2^ = 0.041; *p* = 0.839).

In terms of awareness of the GNRP, 11.29% of respondents actively participated in the program, 83.33% were aware of its existence but were not directly involved, and 4.84% had no prior knowledge of it. Consequently, most respondents evaluated the program based on indirect or observational experience rather than personal participation, which should be considered when interpreting the findings.

## Results

Table 1 presents the distribution of respondents according to the number of years spent working in healthcare. Although the total study sample consisted of 189 participants, complete data on occupational subgroup and years spent working in healthcare were available for 178 respondents; therefore, Table 1 is based on available cases. A significant difference was found between the groups [F(5, 172) = 7.816, *p* < 0.001], with a substantial effect size. The results indicate that nurses spent significantly more years working in healthcare than resident physicians and assistant nurses. The number of years spent working in healthcare was highest among nurses, with a mean of 21.76 years. Among specialist physicians and assistants, the number of years spent working in healthcare was significantly higher than among residents. However, the standard deviation is considered high, with the exception of assistant nurses and residents.

**Table 1:** Number of years spent working in healthcare by occupational role (available cases, *n* = 178).

Position	Mean	N	Standard deviation	Kurtosis	Skewness
Healthcare Assistant	1.4167	6	0.97040	−0.059	0.839
Physician Assistant	17.2667	15	11.76233	−1.484	0.377
Auxiliary Nurse	11.0278	9	13.14846	−1.532	0.850
Registered Nurse	21.7571	70	15.87810	−1.645	0.035
Resident Physician	2.9412	17	1.15762	0.298	0.059
Specialist Physician	19.2705	61	12.93399	−0.768	0.669
Total5	**17**.**5014**	**178**	**14**.**60832**	**−1**.**196**	**0**.**522**

Bold values indicate summary statistics for the total sample.

Attitudes toward individual program elements were assessed using a 4-point Likert scale during the survey (Table 2). Mean values of all examined program elements were relatively high, indicating an overall positive attitude. The highest mean values were observed for professional nursing mentoring (M = 3.65; SD = 0.53), factors supporting the organizational integration of residents (M = 3.66; SD = 0.52), and theoretical continuing education (M = 3.62; SD = 0.55). Standard deviations indicate low response variability (SD = 0.52–0.79).

**Table 2:** Attitudes related to program elements.

Opinions about the graduate nurse residency program	N	Mean	Standard deviation
Nurses working in patient care units monitor and support the training of resident nurses.	187	3.2460	0.58053
The graduate nurse residency program lasts for two years.	186	3.2527	0.76802
The operation of the graduate nurse residency program increases the likelihood of nurse retention.	187	3.2674	0.79186
Nurses working in patient care units welcome resident nurses and willingly assist in their training.	184	3.2772	0.59536
I consider the program to be an innovative initiative that provides a novel opportunity to address critical nursing shortage.	188	3.2979	0.74331
Due to the three-month rotational system, residents become familiar with all patient care units of the hospital.	187	3.4866	0.65054
Psychological mentoring is provided to nurses during the program.	186	3.5645	0.54884
The program includes theoretical continuing education.	184	3.6196	0.55006
Professional mentoring is provided to nurses during the program.	186	3.6505	0.53162
During the two-year program, resident nurses become familiar with a broad range of hospital staff.	188	3.6596	0.51819

Based on the factor analysis, two clearly distinguishable factors were identified, which together explained 61.6% of the total variance. The first factor comprises attitudes related to general support, usefulness, and structural elements of the graduate nurse residency program, while the second factor reflects perceptions of ward-level and day-to-day support, as well as the mentoring environment. A moderately positive correlation was found between the two factors (r = 0.45) (Table 3), indicating that overall evaluation of the program and assessment of ward-level support represent distinct yet structurally related dimensions. This suggests that overall support for the program is partly associated with everyday organizational practices but cannot be explained solely by ward-level experiences. The mean score of the factor measuring general program support was 3.47 (SD = 0.49), while the mean score of the factor measuring ward-level support was 3.26 (SD = 0.54).

**Table 3:** Factor structure of attitudes related to the graduate nurse residency program.

Program item	Factor 1	Factor 2
Professional mentoring of nurses.	0.871	
Psychological mentoring of nurses.	0.787	
Theoretical continuing education during the program.	0.690	
The graduate nurse residency program increases the likelihood of nurse retention	0.677	
The residency program lasts for two years.	0.658	
During the two-year program, resident nurses become familiar with a broader range of hospital staff.	0.654	
Due to the three-month rotational system, residents become familiar with all patient care units of the hospital.	0.588	
I consider the program to be an innovative initiative that offers a novel opportunity to reduce critical nursing shortage.	0.576	
Nurses working in patient care units supervise and support resident nurses.		0.838
Nurses working in patient care units welcome resident nurses and willingly assist in their training.		0.830
Cronbach's *α*	0.872	0.831

A significant difference was observed between physicians and nurses in the evaluation of overall program support. The Welch two-sample t-test indicated that physicians demonstrated significantly higher support than nurses, with a moderately large effect size [t(183.56) = 5.07; *p* < 0.001; Cohen's d = 0.72]. However, no significant difference was found between the groups in the evaluation of ward-level support [t(185) = 1.83; *p* = 0.069; d = 0.27]. Significant differences were observed across occupational roles in the evaluation of overall program support. As variance homogeneity was violated, a Welch one-way ANOVA analysis was conducted, revealing a significant effect [Welch F(5, 30.63) = 6.19; *p* < 0.001; *η*^2^ = 0.14]. Specialist physicians demonstrated significantly higher support than nurses (*p* < 0.001), while differences between other occupational groups were not significant. In the evaluation of ward-level support, no significant differences were detected between positions [F(5, 181) = 0.81; *p* = 0.542; *η*^2^ = 0.02], suggesting that the assessment of everyday ward-level support is similar across professional groups.

Attitudes toward the graduate nurse residency program [Factor 1: Macro-level (program-level) support; Factor 2: Micro-level (ward-based mentoring) support] were examined using one-way multivariate analysis of variance (MANOVA) according to three categories of knowledge about and participation in the program.

Conditions for multivariate analysis of variance were met: the test of equality of covariance matrices showed no significant differences (Box's M test: *p* = 0.531), and the homogeneity of variance was not violated for any of the dependent variables (Levene's tests: *p* = 0.429 and *p* = 0.563, respectively).

The multivariate test did not indicate a significant difference between the groups in the combined pattern of the two factors [Pillai's trace = 0.001; dF (4, 362) = 0.064; *p* = 0.993; partial *η*^2^ = 0.001].

A hierarchical linear regression model was used to analyze overall program support. In the first model, professional role and years spent in healthcare showed significant explanatory power (R^2^ = 0.145; *p* < 0.001); however, the effect of years was not significant. In the second model (Table 4), the inclusion of ward-level support significantly increased the explained variance (*Δ*R^2^ = 0.159; *p* < 0.001) and showed the strongest association with overall program support in the model (*β* = 0.403; *p* < 0.001). The effect of professional role was attenuated in the second model but remained significant (*β* = −0.317; *p* < 0.001). The final model explained 30.42% of the variance in overall program support (R^2^ = 0.304).

**Table 4:** Hierarchical linear regression model explaining overall support for the graduate nurse residency program.

Variable	*β*	p
Profession (0 = physician, 1 = nurse)	−0.317	<0.001
Years spent working in healthcare	−0.088	0.170
Ward-level support	0.403	<0.001

## Discussion

The results of the study support the conclusion that the implementation of the GNRP was perceived favorably in the Hungarian pediatric hospital environment. The results suggest that although the rate of direct participation was relatively low, awareness of the program among healthcare workers was widespread.

Opinions about the GNRP indicate broad support from the professional community, which is crucial for nursing workforce supply (Table 2). This is of particular importance in light of the WHO 2025 report, which emphasizes the global and regional challenges of nursing workforce shortages (4). However, the findings should be interpreted as evidence of perceived acceptance and organizational support rather than evidence of program effectiveness.

An analysis of the latent structure underlying attitudes identified two distinct dimensions: overall support for the program and the perception of day-to-day support within the clinical unit environment. Although substantial differences emerged across professional roles in evaluations of the program as a whole, perceptions of unit-level support were more consistent, suggesting that everyday supportive practices are implemented in a relatively uniform manner at the organizational level (29).

Multiple analyses demonstrate professional role-related differences in the acceptance of the GNRP. Physicians assigned significantly higher ratings to overall program support than nurses, as demonstrated by both independent-samples t-tests and analysis of variance. In the hierarchical regression model, professional role remained associated with overall program support even after the inclusion of ward-level support. This finding may reflect differences in professional socialization and role expectations. Physicians are familiar with residency-type training as part of their own professional pathway, whereas for nurses in the Hungarian context, a structured residency program represents a comparatively novel initiative. At the same time, the more reserved support observed among nurses should not be interpreted as resistance to the program. Rather, nurses may evaluate the program through the lens of everyday clinical workload, potential mentoring responsibilities, emotional labor, staffing pressures, and expectations related to their direct role in supporting resident nurses (11, 12, 20, 21). These factors may make nurses more cautious when evaluating the organizational feasibility of the program, even when they recognize its professional value.

Overall, the results depict a favorable appraisal of several program components. Mean scores were consistently above the midpoint of the scale, indicating that the majority of respondents considered the program to be adequate and worthy of support (Table 2). The relatively low standard deviations reflect a high degree of response homogeneity, pointing to broad consensus among participants. The strongest endorsements were observed for theoretical continuing education, professional and psychological mentoring, and opportunities to expand professional networks. These findings suggest that participants viewed the residency program not merely as a labor market instrument but also as a meaningful opportunity for professional and personal development, a conclusion that is consistent with previous research highlighting competence development and personal satisfaction as key benefits of such programs (30).

The results of the hierarchical regression analysis indicate that differences in attitudes are expressed primarily in their intensity rather than in qualitatively distinct patterns. The inclusion of unit-level support substantially increased the explained variance in overall program support and showed the strongest association with it, while the association with professional role was attenuated but did not disappear entirely. This pattern suggests that evaluations of the program were related mainly to everyday organizational experiences rather than to sharply differentiated attitudinal orientations (31).

With regard to practical implications, the findings suggest that efforts to strengthen acceptance of the program should include attention to the unit-level environment. High ratings assigned to professional and emotional mentoring underscore the pivotal role of these components in perceived program acceptance. Strengthening supportive organizational practices may help ensure that the program is perceived not merely as a structural innovation, but as an integral opportunity for development embedded in everyday work (32). At the same time, the interpretation of role-related differences requires caution. Years spent working in healthcare did not independently explain overall program support in the regression model; therefore, more reserved support among nurses should not be attributed to professional experience itself. Rather, it may be related to the practical implications of nurses' professional role, including anticipated mentoring responsibilities, workload, emotional labor, staffing pressures, and the need to integrate resident nurses into already demanding clinical routines. These issues should be addressed explicitly during program communication and implementation.

Among individual program components, the highest ratings were assigned to the items stating that ‘during the two-year program, resident nurses become familiar with a broad range of hospital staff’ and ‘professional mentoring is provided to nurses during the program’ (Table 2). These findings are consistent with the international literature, which emphasizes that adequate support and structured transition programs significantly contribute to successful adaptation, the development of clinical competencies, and professional integration (17, 19). ‘Emotional mentoring of nurses' also received a high rating, which is particularly noteworthy, as inadequate stress management and lack of support may exacerbate the phenomenon of transition shock during the first year of practice (15, 16, 33).

From a practical perspective, the findings point to several avenues for implementation. The lower average level of support observed among nurses suggests that program communication should place greater emphasis on components that are particularly salient to this group, such as realistic mentor support, workload management, and the institutional resources available for professional mentoring. The high ratings for both professional and emotional mentoring further reinforce the international evidence base, which underscores the central importance of mentoring in transition and residency-type programs (17, 19). In addition, a phased implementation of the program may be beneficial, as it would allow positive experiences to be shared while also acknowledging the workload and role expectations of nurses involved in mentoring.

Based on the results, awareness of or participation in the GNRP showed no detectable relationship with either the overall assessment of the program or the perception of departmental support. This finding should be interpreted in light of the fact that only a small proportion of respondents had direct experience with the program, whereas most respondents evaluated it on the basis of indirect or observational knowledge.

Table 5 summarizes support for the graduate nurse residency program across various variables.

**Table 5:** Summary of results.

Factor	Statistical test	Result	Interpretation
Ward-level support (Factor 2)	Hierarchical regression	β = 0.403, *p* < 0.001	Strongest association with program support
Professional role	Hierarchical regression	β = −0.317, *p* < 0.001	Physicians showed higher support than nurses
Physician vs nurse comparison	Welch t-test	t (183.56) = 5.07, *p* < 0.001, d = 0.72	Large difference favoring physicians
Occupational groups	Welch ANOVA	F (5,30.63) = 6.19, *p* < 0.001, *η*^2^ = 0.14	Specialist physicians showed higher support than nurses
Years in healthcare	Hierarchical regression	β = −0.088, *p* = 0.170	No independent association
Program awareness/participation	MANOVA	Pillai's Trace = 0.001, *p* = 0.993	No independent association
Ward-level support by occupation	ANOVA	F (5,181) = 0.81, *p* = 0.542	No group differences

### Strengths and limitations

One of the major strengths of this study is that, to our knowledge, this is the first GNRP implemented in a hospital setting in Hungary, constituting an innovative initiative that offers a novel opportunity to support the professional integration of newly graduated nurses. The representativeness of the sample in terms of the distribution of occupational roles enhances the internal validity of the findings. Another strength is the examination of the latent structure underlying attitudes toward the program using factor analysis, which identified two dimensions with acceptable reliability indices. Moreover, the multivariate analytical strategy (independent-samples t-tests, analysis of variance, and hierarchical regression) enabled a robust assessment of the observed associations.

At the same time, several limitations should be acknowledged. Data were collected in a single institution that also developed and implemented the GNRP, which restricts the generalizability of the results and raises the possibility of institutional optimism bias. Although the survey was anonymous, employees may have been reluctant to express critical opinions about an employer-sponsored initiative. Furthermore, only a small proportion of respondents had direct experience with the program; therefore, most evaluations reflected indirect or observational knowledge rather than personal participation. The cross-sectional design did not allow for examination of changes in attitudes over time or causal relationships, and the pilot nature of the program means that the findings refer to acceptance and perceived feasibility rather than effectiveness. Future research should therefore incorporate longitudinal follow-up, outcome indicators, and qualitative approaches to provide a more comprehensive understanding of the actual effects of the program.

## Conclusions

This study provides empirical evidence on the acceptance and perceived feasibility of a GNRP in the Hungarian hospital environment. Overall, the results indicate favorable attitudes, particularly toward structured mentoring, organized professional support, and opportunities for competence development. The observed associations suggest that perceived ward-level support and professional role are related to overall program acceptance. However, because of the cross-sectional design, these findings should not be interpreted as evidence of program effectiveness or causal impact. Particular attention should be paid to differences in attitudes between nurses and physicians, as reflected in their perceptions of and support for the program. Exploring the underlying causes of these differences requires further qualitative and longitudinal research. Despite the limitations of the pilot study, the findings support the relevance of further developing and evaluating graduate nurse residency programs (GNRP) in Hungary.

Particular attention should be paid to differences in attitudes between nurses and physicians, as reflected in their perceptions of and support for the program. Exploring the underlying causes of these differences requires further targeted research, using both qualitative and quantitative methods, to better understand potential impacts of interprofessional dynamics, organizational culture, and professional role perceptions on the implementation and sustainability of the program.

Overall, the introduction of the GNRP can be considered a strategically important initiative to support professional socialization and strengthen the long-term stability of the healthcare system. Future studies are needed to assess its effectiveness and long-term outcomes.

## Data Availability

The datasets generated and analyzed during the current study are available from the corresponding author on reasonable request.
